# Identification of Bioactive Components of *Stephania epigaea* Lo and Their Potential Therapeutic Targets by UPLC-MS/MS and Network Pharmacology

**DOI:** 10.1155/2022/3641586

**Published:** 2022-04-27

**Authors:** Xingyu Li, Minyue Li, Zichao Mao, Yue Du, Sylvia Brown, Xiaoyu Min, Ruiqi Zhang, Yun Zhong, Yumei Dong, Zhengjie Liu, Chun Lin

**Affiliations:** ^1^College of Science, Yunnan Agricultural University, Kunming 650201, China; ^2^College of Agriculture and Biotechnology, Yunnan Agricultural University, Kunming 650201, China; ^3^Center of Improvement and Utilization of Characteristic Resource Plants, Yunnan Agricultural University, Kunming 650201, China

## Abstract

*Stephania epigaea*, an important traditional folk medicinal plant, elucidating its bioactive compound profiles and their molecular mechanisms of action on human health, would better understand its traditional therapies and guide their use in preclinical and clinical. This study aims to detect the critical therapeutic compounds, predict their targets, and explore potential therapeutic molecular mechanisms. This work first determined metabolites from roots, stems, and flowering twigs of *S. epigaea* by a widely targeted metabolomic analysis assay. Then, the drug likeness of the compounds and their pharmacokinetic profiles were screened by the ADMETlab server. The target proteins of active compounds were further analyzed by PPI combing with GO and KEGG cluster enrichment analysis. Finally, the interaction networks between essential compounds, targets, and disease-associated pathways were constructed, and the essential compounds binding to their possible target proteins were verified by molecular docking. Five key target proteins (EGFR, HSP90AA1, SRC, TNF, and CASP3) and twelve correlated metabolites, including aknadinine, cephakicine, homostephanoline, and N-methylliriodendronine associated with medical applications of *S. epigaea*, were identified, and the compounds and protein interactions were verified. The key active ingredients are mainly accumulated in the root, which indicates that the root is the main medicinal tissue. This study demonstrated that *S. epigaea* might exert the desired disease efficacy mainly through twelve components interacting via five essential target proteins. EGFR is the most critical one, which deserves further verification by biological studies.

## 1. Introduction

*Stephania epigaea* Lo, belonging to the family Menispermaceae, is an herbaceous liana that primarily grows mainly in limestone hills and is found in Guangdong, Guangxi, Hainan, Yunnan, and Sichuan provinces of China, where it is called “dì bù róng,” “jīn bù huàn,” or “shān wū guī” [1, 2]. Its root tuber has been used as a traditional folk medicine for anti-inflammatory, relieving pain, and sedation to treat cancer, fever, cough, malaria, diarrhea, bellyache, stomachache, and injuries from falls and fractures by local people [3–5]. A total of 40 alkaloids have been identified from the plant since the study of their chemical constituents was first reported in 1975 [6], which are divided into seven categories, including protoberberine-, aporphine-, morphine-, hasubanan-, benzylisoquinoline-, bisbenzylisoquinoline-, and azafluoranthene-type alkaloids, which have been evaluated for biological activity, such as acetylcholinesterase (AChE) inhibitory, cytotoxic, anti-inflammatory, antitumor activities [7–10]. However, these confirmed biological activities do not well explain traditional folk medicine applications of *S. epigaea*. Therefore, to increase our understanding of uncovering the molecular mechanisms of traditional folk medicine applications for *S. epigaea*, the roots, stems, and flowering twigs (described as flowers) of *S. epigaea* were collected, and UPLC-ESI-MS/MS carried out widely targeted metabolomic analysis. Furthermore, a network-based pharmacology study on multiple compounds, multiple targets, and multiple pathways was performed for insight into the medicine activity mechanisms of *S. epigaea*.

## 2. Materials and Methods

### 2.1. Plant Materials

The plants were collected in Dali city and taxonomically identified as *S. epigaea* Lo by Professor Guo Fenggen of Yunnan Agricultural University. The specimens (201703066) were kept in the herbarium of the School of Agronomy and Biotechnology, Yunnan Agricultural University.

### 2.2. Metabolite Extraction

The fresh roots, stems, and flowers of five years were freeze-dried using lyophilizer (Scientz-100F), respectively, and crushed using a mixer mill (MM 400, Retsch) with a zirconia bead for 1.5 min at 30 Hz. The 100 mg powder was extracted overnight with 0.6 mL of 70% methanol at 4°C. Following centrifugation at 10,000 g for 10 min, the supernatant was filtered with a microporous membrane (0.22 *μ*m pore size) and used for UPLC-MS/MS analysis.

### 2.3. UPLC-ESI-MS/MS Analytical Conditions

All samples were analyzed using a UPLC-ESI-MS/MS system (UPLC, Shim-pack UFLC SHIMADZU CBM30A system, https://www.shimadzu.com.cn/; tandem mass spectrometry, MS/MS, Applied Biosystems 4500 Q TRAP, https://www.appliedbiosystems.com.cn/). The analytical conditions were as follows: UPLC: column, Agilent SB-C18 (1.8 *µ*m, 2.1 mm × 100 mm); the mobile phase consisted of solvent A (0.1% formic acid in HPLC grade water) and solvent B (acetonitrile). Sample measurements were performed with a gradient program that employed the starting conditions of 95% A and 5% B. Within 9 min, a linear gradient to 5% A and 95% B was programmed, and a composition of 5% A and 95% B was kept for 1 min. Subsequently, a composition of 95% A and 5.0% B was adjusted within 1 min and then held to 10.00 min to restart the following analysis. The column oven was set to 40°C; the injection volume was 4 *μ*L with an effluent speed of 0.35 mL/min. The effluent was connected to an ESI-triple quadrupole linear ion trap (QTRAP)-MS.

Linear ion trap (LIT) and triple quadrupole (QQQ) scans were acquired on a triple quadrupole linear ion trap mass spectrometer (Q TRAP) and API 4500 Q TRAP UPLC/MS/MS system, equipped with an ESI Turbo Ion-Spray interface, operating in positive and negative ion modes and controlled by Analyst 1.6.3 software (AB Sciex). The ESI source operation parameters were as follows: an ion source, turbo spray; source temperature, 550°C; ion spray voltage (IS), 5500 V (positive ion mode)/-4500 V (negative ion mode); ion source gas I (GSI), gas II(GSII), and curtain gas (CUR) was set at 50, 60, and 30 psi, respectively; and the collision gas (CAD) was high. Instrument tuning and mass calibration were performed with 10 and 100 *μ*mol/L polypropylene glycol solutions in QQQ and LIT modes. QQQ scans were acquired as MRM experiments with collision gas (nitrogen) set to 5 psi. DP and CE for individual MRM transitions were performed with further DP and CE optimizations [11]. A specific set of MRM transitions were monitored for each period according to the metabolites eluted within this period.

### 2.4. Metabolite Analysis

To compare the differences in the metabolites, the mass spectral peaks of each metabolite detected in different samples were corrected to ensure the accuracy of qualitative and quantitative analyses. The mass spectrometry file of each sample was opened with MultiQuant version 3.0.3 software (Sciex, Darmstadt, Germany), and the integration and correction of chromatographic peaks were conducted. The peak area of each chromatographic peak represents the relative levels of the corresponding substances. Based on the MetWare metabolism self-built plant-specific DataBase (MWDB), the qualitative analysis of substances was carried out according to the secondary mass spectrometry information, removing the isotopic signals, repeated signals containing K^+^ ions, Na^+^ ions, and NH_4_^+^ ions, and the signals of other large molecular fragment ions. The metabolites were quantified by triple quadrupole mass spectrometry's multiple reaction monitoring (MRM) model [12]. The UPLC-MS/MS data were processed using Analyst 1.6.3 software (AB Sciex) with default parameters. Quality control samples were prepared by mixing sample extracts and analyzing the repeatability of samples by the same treatment methods. In instrumental analysis, a quality control sample was analyzed every ten samples to monitor the repeatability of the UPLC-MS/MS system over the entire detection process.

### 2.5. Acquisition and Processing for Bioinformatics of Metabolites

Absorption, distribution, metabolism, excretion, and toxicity (ADMET) properties of the metabolites of *S. epigaea* were predicted by using the ADMETlab 2.0 server (https://admetmesh.scbdd.com/) [13], which is a free online platform that facilitates researchers to predict the ADMET and drug-likeness properties of a compound. The two-dimensional (2-D) and three-dimensional (3-D) structures of the metabolites were obtained from PubChem (https://pubchem.ncbi.nlm.nih.gov/). The putative protein targets of the metabolites were retrieved from SwissTargetPrediction (https://www.swisstargetprediction.ch/) [14]. These targets were used to construct the protein-protein interaction (PPI) network through the online tool STRING v.11.0 (https://string-db.org/), and from which the GO [15] and KEGG [16] enrichments results were obtained. Finally, the enriched pathways were used to search for the relevant disease pathways by using the KEGG database (https://www.kegg.jp/) again.

### 2.6. Network Construction and Statistical Analysis

The networks of protein-protein interaction (PPI), compound-target (C-T), target-pathway (T-P), pathway-disease (P-D), and comprehensive network (C-T-P-D) were constructed and visualized using Cytoscape v.3.9.0 (https://cytoscape.org/) [17].

For functional module identification, the two-mode T-P relationships were first transformed into the one-mode target-target (T-T) relationships using Excel2Pajek 5.14 (https://mrvar.fdv.uni-lj.si/pajek/) [18]. Then, the target-pathway-disease (T-P-D) and T-T network were constructed using Gephi v.0.92 (https://gephi.org/), and in which the modularity classes were analyzed and identified using the Louvain algorithm with a resolution of 1.0.32 [19]. In addition, the functional module is evaluated by its contribution score (CS) to a specific disease category that is calculated according to the reference [20, 21], and the module with the highest contribution value is obtained. In this module, the most critical target is evaluated by the protein's integrated centrality (IC) degree calculated by the following equation.

(1) $$\begin{array}{c} {\text{IC}}_{i}=\frac{1}{4}\;\left(\frac{{\text{DC}}_{i}\;-\;{\text{DC}}_{\min}}{{\text{DC}}_{\max}\;-\;{\text{DC}}_{\min}}\;+\;\frac{{\text{BC}}_{i}\;-\;{\text{BC}}_{\min}}{{\text{BC}}_{\max}\;-\;{\text{BC}}_{\min}}\;+\;\frac{{\text{CC}}_{i}\;-\;{\text{CC}}_{\min}}{{\text{CC}}_{\max}\;-\;{\text{CC}}_{\min}}\;+\;\frac{{\text{EC}}_{i}\;-\;{\text{EC}}_{\min}}{{\text{EC}}_{\max}\;-\;{\text{EC}}_{\min}}\right),\;\left(i=1,2,3,4,5,6,\ldots ,I\right), \end{array}$$

where IC_*i*_ refers to the integrated centrality of target *i*; DC_*i*_, BC_*i*_, CC_*i*_, and EC_*i*_ refer to the degree, betweenness, closeness, and eigenvector centrality of target *i*; DC_min_, BC_min_, CC_min_, and EC_min_ refer to the minimum degree, betweenness, closeness, and eigenvector centralities of the functional module; and DC_max_, BC_max_, CC_max_, and EC_max_ refer to the maximum degree, betweenness, closeness, and eigenvector centralities of the functional module. The value of IC ranged from 0 to 1. The higher the IC value of a target, the more important it is in its functional module from the topological perspective.

### 2.7. Molecular Docking Simulation

To confirm the binding affinity of an essential protein target to the metabolites of *S. epigaea*, molecular docking simulation was performed using AutoDock Vina v.1.2.0 (https://vina.scripps.edu/) [22]. The 3D protein structure was downloaded as a pdb file from the PDB database (https://www.rcsb.org/) and uploaded to PyMOL v.2.5.2 (https://pymol.org/2/) to remove water molecules and other ligands from the structure before it was saved as a pdb file [23]. The polar hydrogens and charges were added to the protein structure using MGLTools (https://mgltools.scripps.edu/) and saved as a pdbqt file, and the protein grid box was set to cover up the entire protein molecule with a spacing of 1 angstrom (Å) in MGLTool, and the grid box coordinates were saved as a text file [24].

The 3D metabolite structure was downloaded as an sdf file from PubChem (https://pubchem.ncbi.nlm.nih.gov/) and converted to a pdb file using Open Babel (https://openbabel.org/wiki/Main_Page) [25]. Charges were added, and the torsion tree was constructed using MGLTools before it was saved as a pdbqt file.

The blind docking with the AutoDock Vina [22] was performed where the protein structure in the pdbqt format was set as the receptor, the structure of the metabolite in the pdbqt format was set as the ligand, and the grid box coordinates were copied from the txt file of the protein grid box. Once the docking was performed, the ligand configurations in the protein structure were generated and saved as a pdbqt file. These configurations' corresponding binding free energy changes (ΔE) were calculated and saved as a log.txt file [22]. The visualization of the docking structures was achieved in PyMOL [26] by uploading both protein structure and ligand configurations in the pdbqt format. The images of molecular docking were exported from PyMOL as png files.

## 3. Results

### 3.1. Widely Targeted Metabolite Profiling in Different Tissues

**Figure 1:**
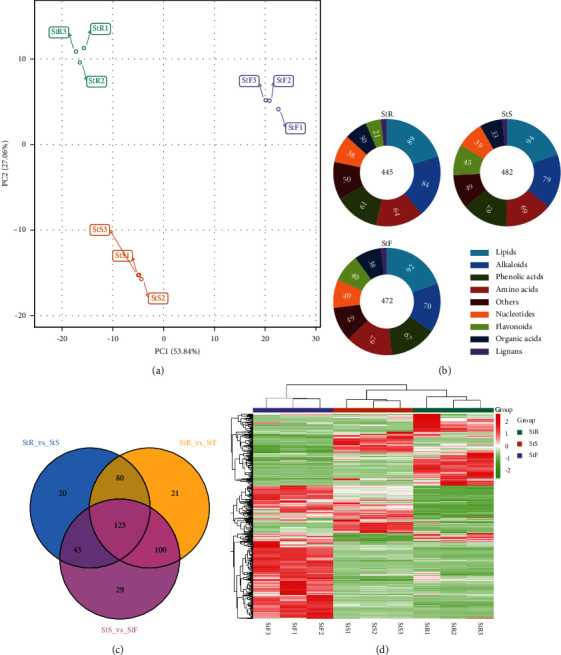
Widely targeted metabolomic profiling identified the metabolites in the tissues of *Stephania epigaea*. (a) PCA score diagram of mass spectrometry data of each group of samples, where StR represents root, StS represents stem, and StF means flower. (b) Differential cumulative distribution of various metabolite categories in the three tissues. (c) Venn diagram of group differences. (d) Differential metabolite cluster heatmap. The cluster tree on the left side of the figure is the differential metabolite cluster tree. Different colors are the relative content. The values are obtained after standardized treatment (red represents high content, and green represents low content).

Through widely targeted metabolomic analysis, 518 metabolites were detected (Table S1) and were categorized into 8 classes of natural compounds such as alkaloids, amino acids, flavonoids, lignans, lipids, nucleotides, organic acids, phenolic acids, and others. Principal component analysis (PCA) by the *R* package of PCAtools (https://github.com/kevinblighe/PCAtools) showed that there were significant chemical differences in the tested samples, indicating that there was an obvious separation trend among the metabolic of the three different tissues (Figure 1(a)). As shown in Figure 1(b), the roots, stems, and flowers contained 445, 482, and 472 metabolites, respectively. The abundance of lipids in each tissue was about 20%. The abundance of alkaloids in each tissue was the second, and there were differences. For example, 84 alkaloids in roots accounted for 18.88%, 79 alkaloids in stems accounted for 16.39%, and 70 alkaloids in flowers accounted for 14.83%. Except for the different content of flavonoids (in roots 4.72%, in stems 9.34%, and in flowers 8.47%), the proportion of other metabolites in each tissue was similar. However, Venn vitalizing [27] of different metabolites in each group showed that there were 268 differential metabolites between root and stem, 297 differential metabolites between flower and stem, and 326 differential metabolites between root and flower, of which 125 differential metabolites existed in three different tissues (Figure 1(c)). Moreover, hierarchical cluster analysis (HCA) of heatmap [28] analysis of metabolite accumulation patterns among different samples also suggested the same trend: the heterogeneity between stems and flowers, and roots and stems was lower than between roots and flowers (Figure 1(d)). Therefore, the characteristics of metabolites in the roots, stems, and flowers of *S. epigaea* were different.

### 3.2. Prediction of ADMET and Drug-Likeness Properties

To gain insight into the pharmacokinetic profile of 518 metabolites of *S. epigaea* and whether they have the potential to become a drug, we used ADMETlab 2.0 to predict its ADMET and drug-likeness properties. The corresponding predicted results are presented in Table S2 (see Supplementary Data). Two hundred metabolites were suggested as putative active compounds because they have relatively fine drug likeness and less toxicity overall, according to the excellent selection criteria of ADMETlab 2.0. [13], e.g., QED score ≥ 0.67, Lipinski < 2violations, Caco − 2 Permeability > −5.15:HIA0 − 0.3, PPB ≤ 90%, QED score ≥ 0.67, QED score ≥ 0.67, QED score ≥ 0.67, Fu ≥ 5%, CL ≥ 5, H − HT ≥ 0 − 0.3, hERG Blockers ≥ 0 − 0.3, F(20%) ≥ 0 − 0.3, F(30%) ≥ 0 − 0.3Ames Toxicity ≥ 0 − 0.3, Fsp^3^ ≥ 0.42, MCE-18 > 45, PAINS not 0, and Golden Triangle 0 violations.

### 3.3. Target Identification and Bioinformatic Mining of Metabolites

The 200 putative active compounds were conducted using target prediction through the Swiss target prediction online software [14]. A total of 393 proteins were retrieved as putative targets for species limited to “*Homo sapiens*,” with a probability greater than 0.1. The results are shown in Table S3 (see Supplementary Data).

The 393 putative protein targets were introduced into the STRING database for bioinformatic mining, and the organism was selected as “*Homo sapiens*,” setting the minimum required interaction score as confidence score >0.4. Then, KEGG enrichment data were downloaded for further network pharmacology research, in which a total of 165 pathways were obtained with the criteria of false discovery rate (FDA) <0.05 [29]. The results are shown in Table S4 (see Supplementary Data). A total of 46 KEGG disease entries were associated with 62 pathways of human diseases and one pathway of environmental information processing and signal transduction that related the nephrotic syndrome with urinary system disease (Table S5). A total of 167 of the 393 putative targets were enriched in these disease pathways. These diseases are categorized into infectious, cancer, neurodegenerative, urinary system, metabolic, mental, immune system, cardiovascular, drug resistance, and substance dependence.

### 3.4. Network Construction and Analysis

**Figure 2:**
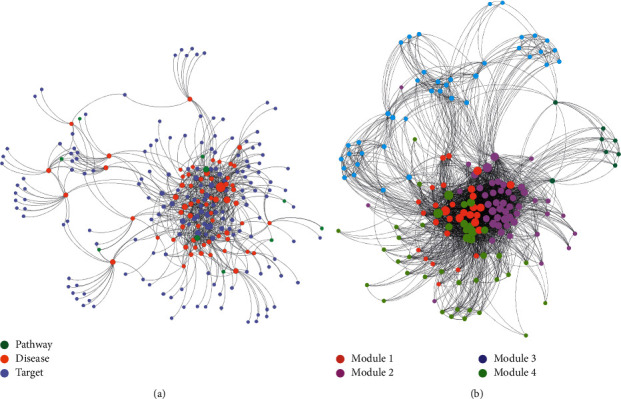
(a) The target-pathway-disease interaction network and (b) the target-(pathway)-target interaction network with modularity partition by Gephi with Louvain algorithm, where the nodes were targets and the edges were the shared pathways of these targets.

A network of interrelated targets, pathways, and disease categories (T-P-D) was constructed (Figure 2(a)), which consisted of 240 nodes, *including* 167 targets, 63 pathways, 10 related diseases, and 796 edges. As shown in Figure 2(a), a target protein in the constructed network was connected to either one or multiple pathways, which related to either one or multiple disease types. The T-T network was constructed through the corresponding relationships between targets and pathways to extract the relationship among the targets. The edges of the network represent the common pathways between every two targets. Through a set of shared pathways, the functional modules of the target are determined. The targets related to the same pathways have a similar biological function (Figure 2(b)). Five functional modules (modules 1–5) were identified, where module 1 consisted of 48 targets (28.74% of total targets), module 2 consisted of 30 targets (17.96%), module 3 consisted of 41 targets (24.55%), module 4 consisted of 40 targets (23.95%), and module 5 consisted of 8 targets (4.79%).

**Figure 3:**
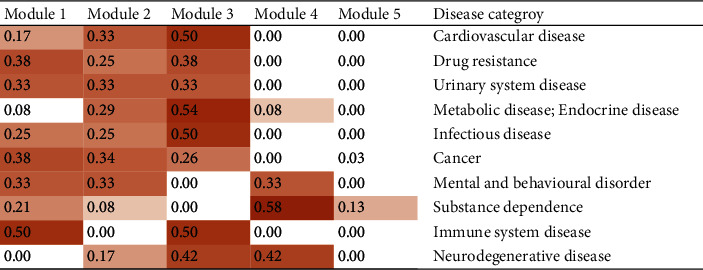
The contribution scores (CSs) of each module to various diseases.

The contribution of each functional module to a particular disease category can be evaluated using the contribution score (CS) of each module. The CSs of the five functional modules to 10 disease categories are calculated in Figure 3. The sum of the CSs of five modules to a disease category was 1 unity, and the larger the CS value of a module, the greater the module's contribution to a disease became. The targets in modules 1–3 were extracted to calculate the integrated centrality (IC) degree to mine the most important target. The IC values were determined and are shown in Table S6 (see Supplementary Data). Five targets with higher IC values (˃0.8) were suggested as the most important targets in the functional module, which were EGFR [30], SRC [31], TNF [32], CASP3 [33], and HSP90AA1 [34], that are all close relative to oncogenesis, cell growth, and immunomodulation.

### 3.5. Molecular Docking Verification

**Figure 4:**
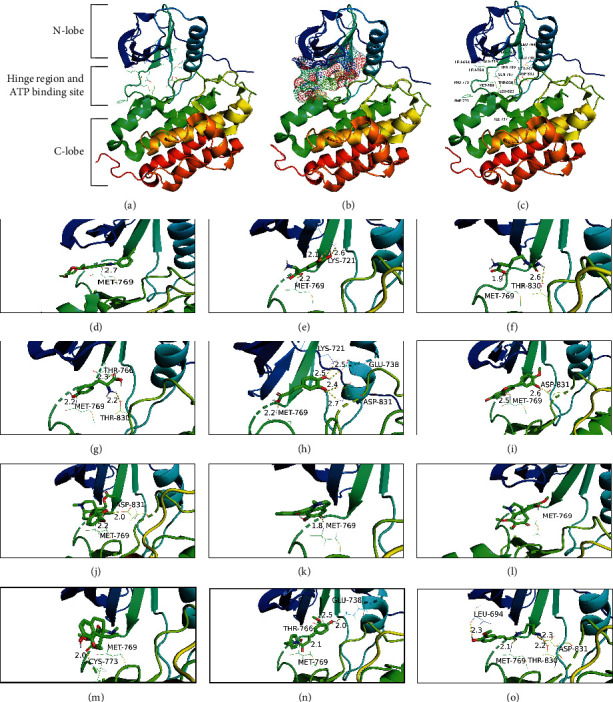
The cartoon diagram of EGFR structure and interactive maps of compounds inside its active site. (a) The overall structure of EGFR (PDB ID: 1M17). (b) The ATP-binding pocket of EGFR is shown as mash surface. (c) Amino acid regions in the active site of EGFR. Molecular interactions of EGFR with (d) erlotinib, (e) 3, 4-dihydroxy-DL-phenylalanine, (f) citrulline, (g) pratensein, (h) tyrosine, (i) homostephanoline, (j) feruloyltyramine, (k) N-feruloylputrescine, (l) L-phenylalanine, (m) caffeic acid, (n) N-methylliriodendronine, and (o) aknadinine. The protein structures were shown in the rainbow-colored cartoon. The amino acid residues at the active sites were shown as colored lines with names and sequence numbers. The hydrogen bonds were shown as yellow dashed lines with distance values in angstrom. The compound structures were shown as colored sticks. Carbon atoms and carbon-carbon bonds were green colored, oxygen atoms were red colored, hydrogen atoms were grey colored, and nitrogen atoms were blue colored.

Twelve compounds correlated to the five most important targets were extracted, and their interaction was further verified by molecular docking simulation. The binding affinity of a ligand-target complex was evaluated by the binding energy change (ΔE), where a more negative binding energy value indicates a stronger binding affinity or a greater binding constant for the formation of the ligand-target complex.  Table 1 shows the binding affinity energies of the 12 compounds to the five essential targets, which had values ranging from −9.1 kcal/mol to −4.9 kcal/mol. The binding energy of ≤−5.0 kcal/mol indicates the strong binding between a ligand and its target [35–37]. The strongest binding was observed between N-methylliriodendronine and SRC with a −9.1 kcal/mol binding energy. Since the stronger the binding affinity of a ligand to its protein target, the higher the potency of the ligand, the binding affinity data can guide us to select the proper ligand-target pairs from each functional module for experimental validation of the efficacy of compounds aimed at illnesses and therapeutic outcomes.  Table 1 also shows that EGFR is a common target of 11 compounds, suggesting the centrality of EGFR in *S. epigaea* treatment of diseases. To explore the possible binding conformation, a molecular docking simulation was performed on the ATP-binding pocket of EGFR (1M17). Analysis of the binding mode of the cocrystallized ligand (erlotinib) at the ATP-binding site of the EGFR revealed that the EGFR tyrosine kinase domain consists of a hydrophobic pocket composed of D831, T830, L820, F771, P770, M769, L768, Q767, L764, MET742, M741, K721, E738, and L694 (Figures 4(a)–4(c)). As shown in Figures 4(d)–4(o), most of the docked metabolites formed a remarkable interaction binding mode with the EGFR active sites. It can be noticed that all derivatives bind to the key amino acid M769 with hydrogen bonds like the original ligand (erlotinib). The amino acid M769 assists in anchoring the ligands to direct them to the hydrophobic pocket of EGFR through hydrogen bond formation.

## 4. Discussion and Conclusions

**Figure 5:**
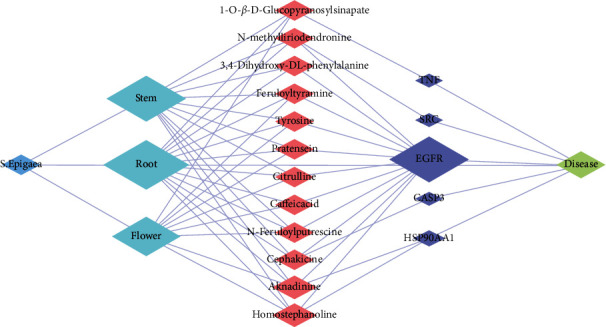
Integrated network for mechanisms of traditional folk medicine applications for *S. epigaea*. Each column represents plants, tissues, metabolites, targets, and diseases from left to right.

Previously mentioned that the medicinal plant *Stephania epigaea* Lo traditionally was used to treat fever, cough, malaria, diarrhea, bellyache, injuries from falls, and fracture [3, 4] and newly discovered activities of antiproliferative/anticancer, immunomodulating, and apoptosis [7, 38]. Our metabolomic analysis shows that 518 metabolites were detected from the root, stem, and flowers of *S. epigaea* through widely targeted metabolomics analysis. Among the metabolites, 200 were selected as putative active compounds because they have relatively fine drug likeness and less toxicity overall, and 393 putative proteins were predicted as targets of those selected compounds. Through PPI analysis, enrichment and analysis of both GO and KEGG, correlated analysis of richening pathway-associated diseases, various biological network construction and analysis, and finally, five essential protein targets and twelve key metabolites were identified and verified by molecular docking simulation to be associated with traditional folk medicine applications of *S. epigaea* (Table 1).  Figure 5 demonstrates that *S. epigaea* exerted its pharmacological effects on humans through multicomponent acts via five essential protein targets to exhibit the desired disease efficacy. It is noteworthy that EGFR is the common target of most alkaline and phenolic compounds.

Furthermore, molecular docking found that these compounds can perfectly combine with EGFR and form hydrogen bonds with the critical amino acid residue of M769, like the original ligand (erlotinib) [39]. EGFR is present on the surface of cells involved in cell growth. The binding of those chemicals to modulate EGFR kinase activity may prevent cancer cells from growing or promoting normal cell growth by recovering damaged tissues or organs from injury of falling or fracture. Thus, some inhibitors of EGFR kinase, such as erlotinib, afatinib, and osimertinib, are used for cancer treatment [40–42]. The chemicals of both cephakicine and N-methylliriodendronine can bind to EGFR, while cephakicine can bind to CASP3 and N-methylliriodendronine can dock SRC, indicating that those compounds can simultaneously affect the regulation of multiple pathways to suppress the caner or tumor initiations and development [43, 44]. We also found that both homostephanoline and aknadinine can bind EGFR and HSP90AA1, the latter, as a molecular chaperon, responding to cancer cells to support folding and activating oncoproteins, including many kinases and transcription factors for cell growth and proliferating, so chemicals modulate Hsp90 activities working as a buffer for these regulators' activity. Therefore, this study provides a base for drug discovery of those four compounds and analogies for cancer/tumor treatment based on further biological and pharmaceutical studies.

**Figure 6:**
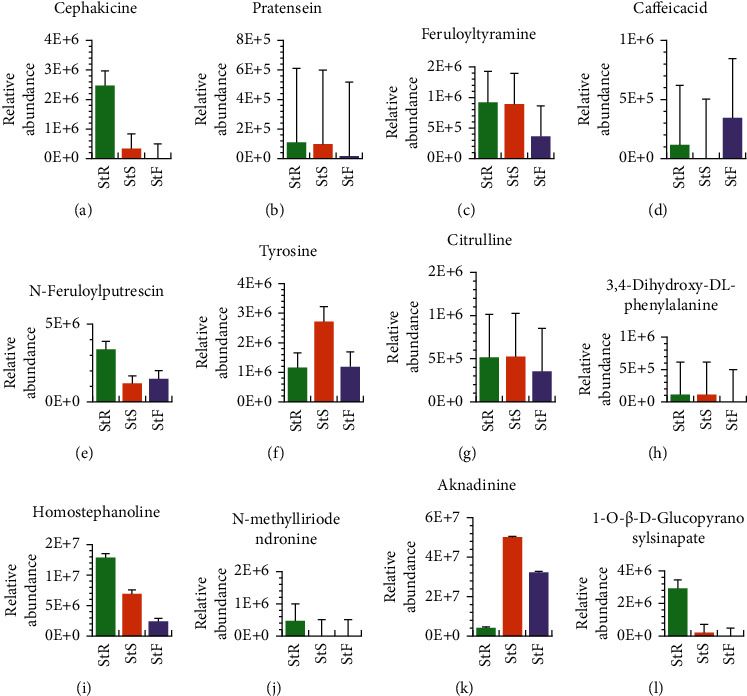
Accumulation of twelve compounds in the three tissues, where StR represents root, StS represents stem, and StF means flower.

The PCA showed significant chemical differences in the tested samples, indicating an obvious separation trend between the metabolites of the three different tissues (Figure 1), and the qualitative and quantitative of the twelve key active components identified are different in the three tissues. As shown in Figure 6, the root contains all twelve active ingredients. Except for compounds caffeic acid Figure 6(d), tyrosine Figure 6(f), and aknadinine Figure 6(k), the content of the rest is the highest in the three tissues, while the stem is less in caffeic acid Figure 6(d). Compounds cephakicine Figure 6(a), 3, 4-dihydroxy-DL-phenylalanine Figure 6(h), N-methylliriodendronine Figure 6(j), and 1-O-*β*-D-glucopyranosyl sinapate Figure 6(l) are missing in flowers. These results are just consistent with the application description of traditional medicine that its root tuber has been used as a traditional folk medicine for some diseases by local people.

## Figures and Tables

**Table 1:** The molecular docking binding affinities (kcal/mol) of core targets and their ligands.

No.	Compounds	PubChem CIDs^a^	Target names	PDB IDs^b^	Uniprot IDs^c^	BAs^d^
1	Cephakicine	15968782	CASP3	1NME	P42574	−5.9
	Cephakicine	15968782	EGFR	1M17	P00533	−6.6
2	Pratensein	5281803	EGFR	1M17	P00533	−8.4
3	Feruloyltyramine	5280537	EGFR	1M17	P00533	−7.1
4	Caffeic acid	689043	EGFR	1M17	P00533	−5.9
5	N-Feruloylputrescine	5281796	EGFR	1M17	P00533	−5.9
6	Tyrosine	6057	EGFR	1M17	P00533	−5.5
7	Citrulline	9750	EGFR	1M17	P00533	−4.9
8	3, 4-Dihydroxy-DL-phenylalanine	836	EGFR	1M17	P00533	−5.5
9	Homostephanoline	627343	EGFR	1M17	P00533	−6.4
	Homostephanoline	627343	HSP90AA1	6CEO	P07900	−5.2
10	N-Methylliriodendronine	135474262	EGFR	1M17	P00533	−8.7
	N-Methylliriodendronine	135474262	SRC	4K11	P12931	−9.1
11	Aknadinine	159966	EGFR	1M17	P00533	−7.4
	Aknadinine	159966	HSP90AA1	6CEO	P07900	−5.8
12	1-O-*β*-D-Glucopyranosyl sinapate	5280406	TNF	6OOY	P01375	−6.9

^a^PDB ID, protein identifier in protein data bank. ^b^PubChem CID, the compound identifier in PubChem database. ^c^UniProt ID, protein name identifier in UniProtKB. ^d^BAs, binding affinity (kcal/mol).

## Data Availability

The datasets generated and analyzed during the current study were uploaded to the manuscript as Supplementary Materials.

## Supplementary Materials

Supplementary Materials Table S1: the metabolites from roots, stems, and flowers of *S. epigaea*. Table S2: the ADMET properties of metabolites of *S. epigaea*. Table S3: the predicted targets of metabolites of *S. epigaea*. Table S4: the pathway enrichment for the targets of metabolites from *S. epigaea*. Table S5: the diseases correlated with the pathway that enriched two targets of metabolites from *S. epigaea* and associated with human diseases. Table S6: the IC of targets from selected functional modules.
